# A multicenter study on the quantification of liver iron concentration in thalassemia patients by means of the MRI T_2_^*^ technique

**DOI:** 10.3389/fmed.2023.1180614

**Published:** 2023-05-19

**Authors:** Fengming Xu, Yuzhao Peng, Hanhong Xie, Bumin Liang, Gaohui Yang, Fanyu Zhao, Yu Liu, Peng Peng

**Affiliations:** ^1^Department of Radiology, The First Affiliated Hospital of Guangxi Medical University, Nanning, Guangxi, China; ^2^School of International Education, Guangxi Medical University, Nanning, Guangxi, China; ^3^Department of Hematology, The First Affiliated Hospital of Guangxi Medical University, Nanning, Guangxi, China; ^4^Department of Radiology, People's Hospital of Guangxi Zhuang Autonomous Region, Nanning, Guangxi, China; ^5^Department of Radiology, The Affiliated Tumor Hospital of Guangxi Medical University, Nanning, Guangxi, China; ^6^NHC Key Laboratory of Thalassemia Medicine, Guangxi Medical University, Nanning, Guangxi, China

**Keywords:** thalassemia, magnetic resonance imaging, iron overload, liver iron concentration, equation

## Abstract

**Objective:**

To investigate the feasibility and accuracy of quantifying liver iron concentration (LIC) in patients with thalassemia (TM) using 1.5T and 3T T_2_^*^ MRI.

**Methods:**

1.5T MRI T_2_^*^ values were measured in 391 TM patients from three medical centers: the T_2_^*^ values of the test group were combined with the LIC (LIC_F_) provided by FerriScan to construct the curve equation. In addition, the liver 3T MRI liver T_2_^*^ data of 55 TM patients were measured as the 3T group: the curve equation of 3T T_2_^*^ value and LIC_F_ was constructed.

**Results:**

Based on the test group LIC_F_ (0.6–43 mg/g dw) and the corresponding 1.5T T_2_^*^ value, the equation was LIC_F_ = 37.393${\text{T}}_{2}{}^{*\wedge }$(−1.22) (R^2^ = 0.971; *P* < 0.001). There was no significant difference between LIC_e − 1.5T_ and LIC_F_ in each validation group (*Z* = −1.269, −0.977, −1.197; *P* = 0.204, 0.328, 0.231). There was significant consistency (Kendall's *W* = 0.991, 0.985, 0.980; all *P* < 0.001) and high correlation (r_s_ = 0.983, 0.971, 0.960; all *P* < 0.001) between the two methods. There was no significant difference between the clinical grading results of LIC_e − 1.5T_ and LIC_F_ in each validation group (χ^2^ = 3.0, 4.0, 2.0; *P* = 0.083, 0.135, 0.157), and there was significant consistency between the clinical grading results (Kappa's K = 0.943, 0.891, 0.953; *P* < 0.001). There was no statistical correlation between the LIC_F_ (≥14 mg/g dw) and the 3T T_2_^*^ value of severe iron overload (*P* = 0.085). The LIC_F_ (2–14 mg/g dw) in mild and moderate iron overload was significantly correlated with the corresponding T_2_^*^ value (r_s_ = −0.940; *P* < 0.001). The curve equation constructed from LIC_F_ and corresponding 3T T_2_^*^ values in this range is LIC_F_ = 18.463T_2_^*^∧^(−1.142)^ (R^2^ = 0.889; *P* < 0.001). There was no significant difference between LIC_F_ and LIC_e − 3T_ in the mild to moderate range (Z = −0.523; *P* = 0.601), and there was a significant correlation (r_s_ = 0.940; *P* < 0.001) and significant consistency (Kendall's *W* = 0.970; *P* = 0.008) between them. LIC_e − 3T_ had high diagnostic efficiency in the diagnosis of severe, moderate, and mild liver iron overload (specificity = 1.000, 0.909; sensitivity = 0.972, 1.000).

**Conclusion:**

The liver iron concentration can be accurately quantified based on the 1.5T T_2_^*^ value of the liver and the specific LIC-T_2_^*^ curve equation. 3T T_2_^*^ technology can accurately quantify mild-to-moderate LIC, but it is not recommended to use 3T T_2_^*^ technology to quantify higher iron concentrations.

## 1. Introduction

The liver is one of the major iron storage organs; LIC reflects the total iron load, which is an important clinical indicator for clinical monitoring, evaluation, and treatment of iron overload (1). Although the actual liver iron concentration provided by liver biopsy serves as the “gold standard” for clinical indicators, most scholars and medical centers prefer to use non-invasive MRI technology for LIC monitoring because biopsy provides only small samples and has the disadvantages of invasiveness and poor repeatability (2). The LIC (LIC_F_), based on the MRI T_2_/R_2_ (1000/T_2_) technique and reported by FerriScan (Resonance Health Limited, Burswood, WA, Australia), has been certified by the Food and Drug Administration (FDA) of the United States and has high reliability (3). However, there are many limitations to this technique (4): it requires patient-related MRI T_2_/R_2_ data to be sent to FerriScan for off-site post-processing and analysis. The off-site sending of patient data requires approval from the relevant center and involves time costs that will prolong the time to obtain LIC results. The additional cost of the analysis will also increase the cost of LIC monitoring at one time, which results in the use of FerriScan technology for liver iron quantification being limited to a few large medical centers, and the possibility of regular or long-term LIC monitoring in patients being greatly reduced.

The T_2_^*^ technique, based on the gradient recalled echo (GRE) imaging sequence of MRI has been established as a non-invasive standard for quantifying tissue iron levels (5–7). Many centers have been using the T_2_^*^ relaxation method and corresponding software technology to measure organ relaxation parameters, such as T_2_^*^ and R_2_^*^ (1000/T_2_^*^) values, to indirectly obtain the estimated value of organ iron concentration (8). Some studies have explored the relationship between liver 1.5T T_2_^*^/R_2_^*^ and LIC in patients with iron overload and constructed the corresponding LIC-T_2_^*^/R_2_^*^ curve equation (8–13). However, the equations constructed in these studies, which were partly based on liver iron concentrations obtained from small biopsy samples, have not been validated in multiple centers, and their reliability needs to be verified. Moreover, most of the current studies in this area are based on 1.5T MRI, while the studies based on 3T MRI are few and limited. many studies only discuss the correlation between 3T T_2_^*^/R_2_^*^ and LIC and whether the diagnosis is liver iron overload, but there is less analysis of the cutoff value of clinical classification of mild, moderate, and severe iron overload (14, 15).

The aim of this study was to investigate the relationship between liver 1.5T, 3T T_2_^*^ values and LIC_F_ in thalassemia (TM) patients based on large sample size and multicenter data and also to investigate the feasibility, reliability, and accuracy of 1.5T and 3T MRI T_2_^*^ techniques in quantifying LIC in TM patients.

## 2. Materials and methods

### 2.1. Research data

Liver 1.5T MRI T_2_^*^ data of thalassemia patients from three medical centers from January 2014 to June 2022 were retrospectively analyzed: 273 patients from the First Affiliated Hospital of Guangxi Medical University (Center 1), 54 from the Guangxi Zhuang Autonomous Region Ethnic Hospital (Center 2), and 64 from the Guangxi Medical University Affiliated Tumor Hospital (Center 3). In total, 13 patients underwent 1.5T MRI T_2_^*^ liver scans at three centers (within 24 h of the same patient being scanned at different centers). In addition, 3T liver MRI T_2_^*^ data of 55 TM patients from center 1 were collected. The inclusion criteria were: (1) patients diagnosed with thalassemia by genetic diagnosis, with a history of regular or irregular blood transfusion; (2) age ≥ 9 years old; (3) MRI for thalassemia was performed with liver T2 (as required by FerriScan) and T_2_^*^ sequences. Exclusion criteria were: (1) the image data artifacts were large and did not meet the measurement requirements; (2) patients were complicated with other chronic liver diseases or neoplastic diseases. This study was conducted in accordance with the principles of the Declaration of Helsinki and approved by the Ethics Committee of the First Affiliated Hospital of Guangxi Medical University (2022-E457-01).

### 2.2. MR scanning methods

1.5T data: a Siemens 1.5T MRI scanner (MAGNETOM Avanto Fit & Altea, Siemens Healthcare, Erlangen, Germany), a Philips 1.5T MRI scanner (Achieva, Philips Medical Systems, Best, Netherlands), and the body coil were used. The images reported by FerriScan use a free-breathing two-dimensional multilayer spin-echo pulse sequence: rollover angle = 90°, repeat time (TR) = 1,000 ms, echo time (TE) = 6.0, 9.0, 12.0, 15.0, 18.0 ms, matrix = 256 × 256 mm^2^, layer thickness = 5 mm, FOV = 400 mm × 400 mm. The scan time was 15 min. GRE scan sequence: the same level above the hepatic hilum was scanned with one breath hold at the end of exhalation. rotation angle = 20°, TR = 200.00 ms, TE = 1.29, 2.35, 3.43, 4.6, 5.68, 6.85, 7.93, 9.1, 10.18, 11.35, 12.43, 13.6 ms, matrix = 256 × 256 mm^2^, layer thickness = 10 mm, FOV = 400 mm × 400 mm.

3T data: a Siemens 3T MRI scanner was used (Verio, Siemens Healthcare, Erlangen, Germany). TR = 200 ms, TE = 0.97, 2.38, 3.79, 5.20, 6.61, 8.02, 9.43, 10.84, 12.25, 13.66, 15.07, 16.48 ms; Rotation angle = 20°, matrix = 64 × 128 mm^2^, layer thickness = 10 mm, FOV = 200 mm × 400 mm.

### 2.3. Data measurement and analysis

**Figure 1:**
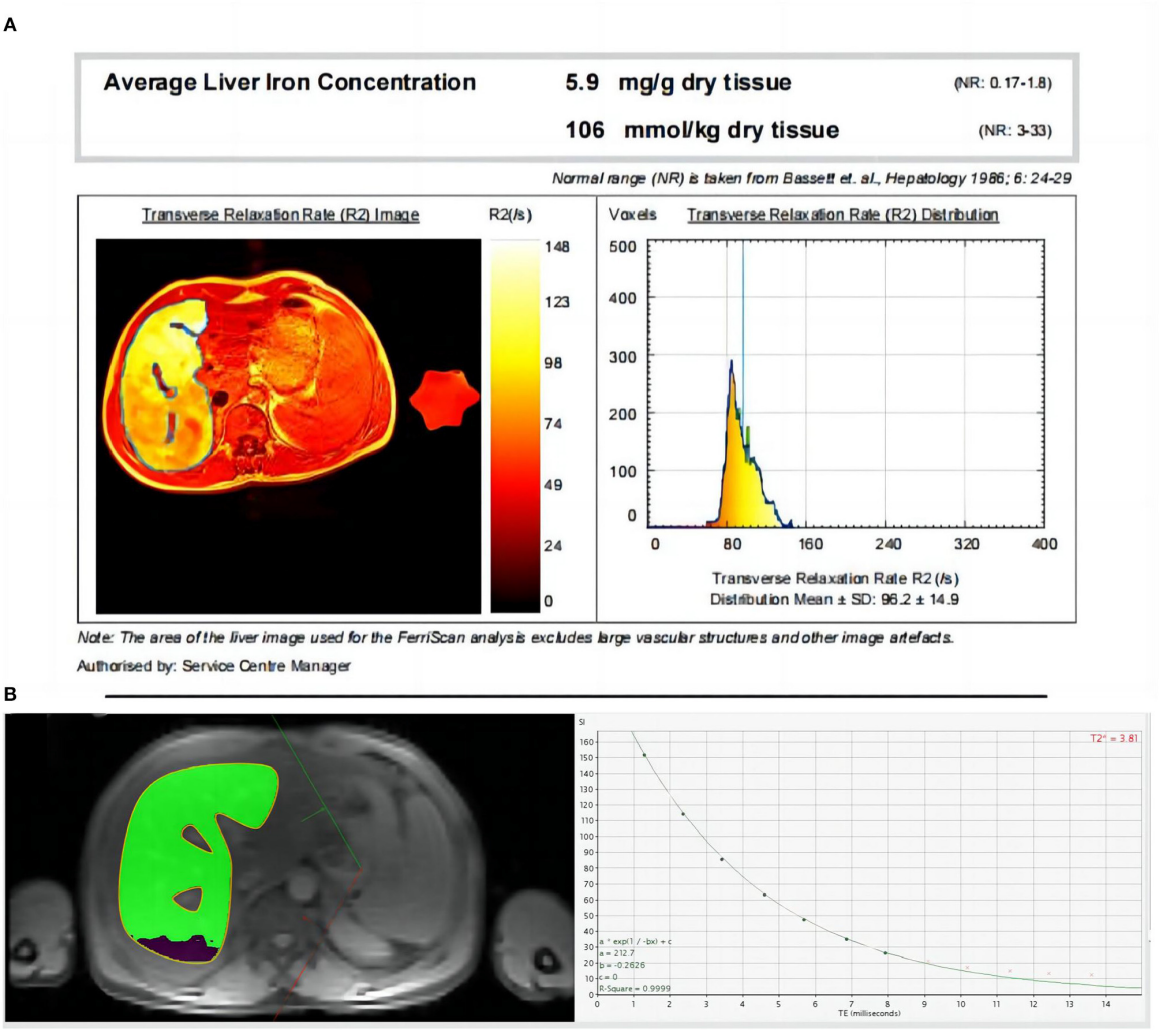
**(A, B)** A 35-year-old male thalassemia patient with an LIC_F_ of 5.9 mg/g dw **(A)** and mild iron overload in the liver; CMRtools showed a mean T_2_^*^ of 3.81ms (the first seven echo signal intensities were included in the fitted curve, and the red “ × ” indicates the echo signal intensities of the removed offset curve) with an R^2^ of 0.9999 **(B)**.

**Figure 2:**
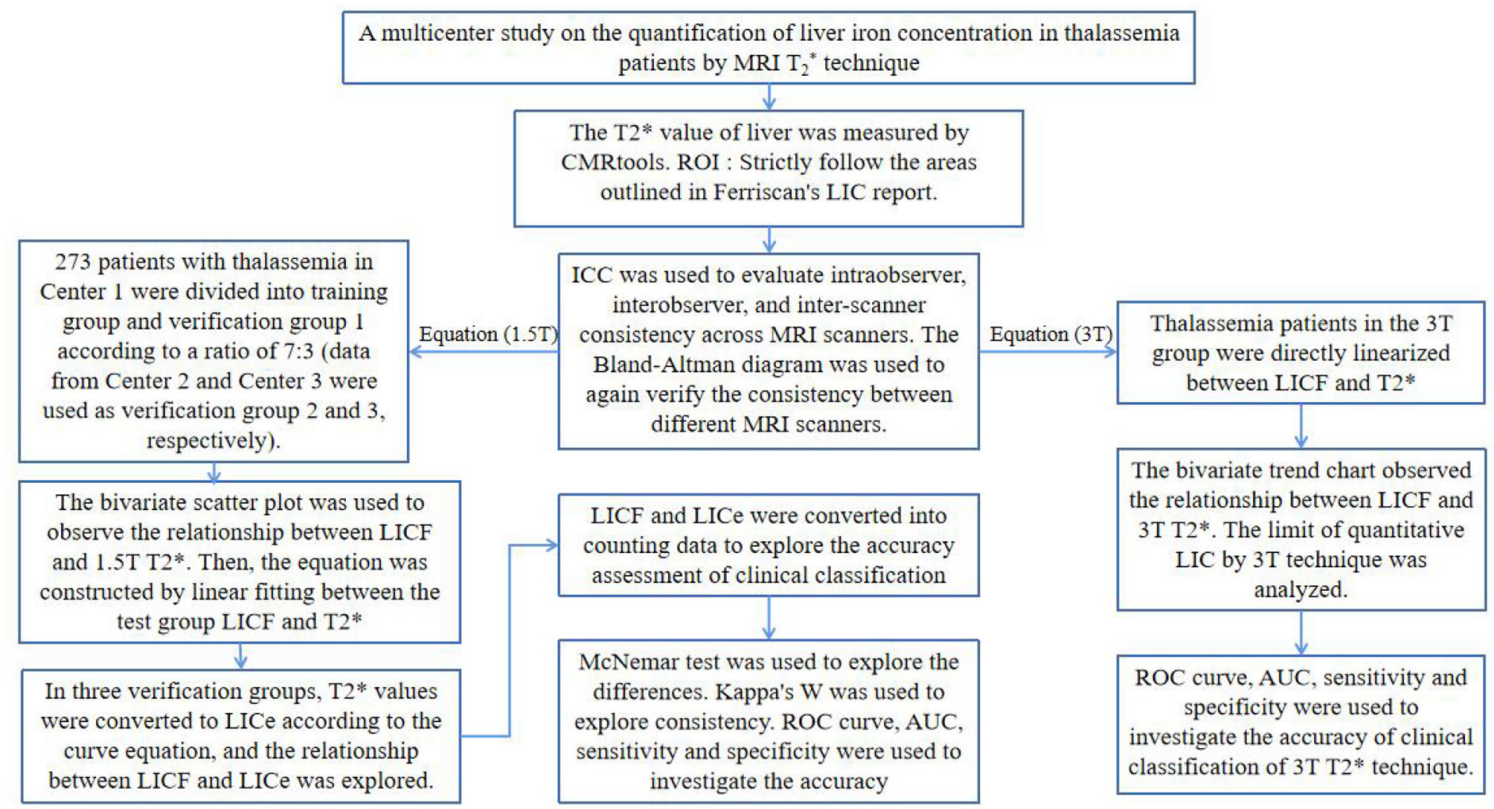
The overall technology roadmap.

The 1.5T T2 image data was sent to FerriScan for post-processing and analysis. The LIC_F_ used in the study was obtained from the final FerriScan report (Figure 1A). The overall technical procedure of this study is shown in Figure 2.

All T_2_^*^ image data were measured using CMRtools (CMRtools/Thalassemia Tools 2014, Cardiovascular Imaging Solutions, London, UK). Measurement procedure: T_2_^*^ image data were exported from the PACS system and imported into a personal computer with CMRtools software installed. The “Thalassemia” function of CMRtools was used to draw a roughly similar ROI according to the range of liver levels measured by FerriScan, avoiding the visible intrahepatic vessels and bile ducts at the same liver level. The delineated ROIs and the fitted T_2_^*^ values were then displayed in the post-processing software. The truncation method (14) was used to reject signal intensity values (SI) that deviated from the fitted curve one by one, and the T_2_^*^ value was recorded when the goodness of fit (R^2^) ≥ 0.98 (Figure 1B).

### 2.4. Statistical methods

SPSS 26.0 statistical software package was used for statistical analysis. All test results were statistically analyzed according to the test significance α = 0.05. MedCalc 19.8.0 statistical software was used to analyze the consistency of T_2_^*^ values measured by different MRI scanners.

The 1.5T data of 273 patients from center 1 were divided into 191 patients in the test group and 82 patients in the validation group 1 according to the ratio of 7:3 by random number method. The 1.5T data of 54 patients from Center 2 were used as validation group 2. The 1.5T data of 64 patients from Center 3 were used as validation group 3. The 3T data of 55 cases from Center 1 were taken as the 3T group (3T data were only self-verified due to a small amount of data and were not grouped).

The intraclass correlation coefficient (ICC) was used, according to the ratio of 7:3. The 1.5T T_2_^*^ image data of 50 randomly selected patients from the test group (*n* = 35) and validation group 1 (*n* = 15) were measured to evaluate intra-observer and inter-observer agreement (independently performed by two radiologists with 5 years of experience in abdominal radiology diagnosis). The intra-observer ICC was calculated by comparing the T_2_^*^ values measured by observer A twice. The inter-observer ICC was calculated by comparing the T_2_^*^ values measured by observer B with the T_2_^*^ values measured by observer A. The ICC between the different MRI scanners was calculated by comparing the 1.5T T_2_^*^ values of 13 patients at three centers. “Two-way random” was selected for “model” and “absolutely consistent” was selected for “type.” When ICC > 0.75 and *P* < 0.05, the measured T_2_^*^ values were considered to be highly consistent. The remaining T_2_^*^ value measurements were performed by observer A. Bland–Altman plots were used to analyze the consistency of the 1.5T T_2_^*^ values of 13 patients measured by different MRI scanners.

The age, LIC_F_, and the measured liver T_2_^*^ values of each group did not follow the normal distribution by the normality test (*P* < 0.05). The interquartile range (P_25%_, P_75%_) and median (M) were used as statistical descriptors. By curve fitting, the calibration curve equation was constructed between the T_2_^*^ value of the test group and the LIC_F_ of the 3T group. The T_2_^*^ values in each validation group and the 3T group were converted into LIC_e − 1.5T_ and LIC_e − 3T_ by the constructed curve equation. Both LIC_e − 1.5T_ and LIC_e − 3T_ did not conform to the normal distribution (*P* < 0.05). The Wilcoxon signed-rank test was used to examine the difference, and *P* > 0.05 was considered as no significant difference. Kendall's *W* coefficient was used to examine consistency. If the consistency coefficient *W* > 0.75 and *P* < 0.05, it indicated a high degree of consistency. Spearman rank correlation analysis was used to examine the correlation; if the correlation coefficient of |*r*_*s*_| > 0.75 and *P* < 0.05, it indicated a high correlation.

LIC_e_ and LIC_F_ were graded according to the severity of clinical liver iron overload, which was divided into normal (< 1.8 mg/g dw), mild (1.8–7.0 mg/g dw), moderate (7.0–14.0 mg/g dw), and severe (>14.0 mg/g dw) liver iron overload (12). The McNemar test was used to examine the difference in clinical grading results. When *P* > 0.05, there was no significant difference between the two. Kappa's coefficient (Kappa's K) was used to examine the consistency of clinical grading results. If K > 0.75 and *P* < 0.05, the two were highly consistent. With the LIC_F_ grading results as the reference standard, the area under the curve (AUC), sensitivity, and specificity of the receiver operating characteristic curve (ROC) was used to evaluate the accuracy of the LIC_e − 1.5T_ and LIC_e − 3T_ clinical classification results in each validation group. The Youden index was used to evaluate the authenticity of the clinical classification results, and the cutoff values of different clinical classifications were recorded when the Youden index was maximum.

## 3. Results

### 3.1. Basic data information

In Center 1, there were 152 male subjects (55.68%) and 121 female subjects (44.32%), aged from 9 to 49 years (M = 19.00, P_25%_ = 12.00, P_75%_ = 27.00). In Center 2, there were 19 male subjects (35.19%) and 35 female subjects (64.81%), aged from 9 to 47 years (M = 13.00, P_25%_ = 12.00, P_75%_ = 21.25). In Center 3, there were 42 male subjects (65.63%) and 22 female subjects (34.37%), aged from 10 to 63 years (M = 15.50, P_25%_ = 11.00, P_75%_ = 25.00). In the 3T group, there were 33 males (60.00%) and 22 females (40.00%), aged from 9 to 25 years (M = 13.00, P_25%_ = 11.00, P_75%_ = 14.00).

### 3.2. Consistency analysis

**Figure 3:**
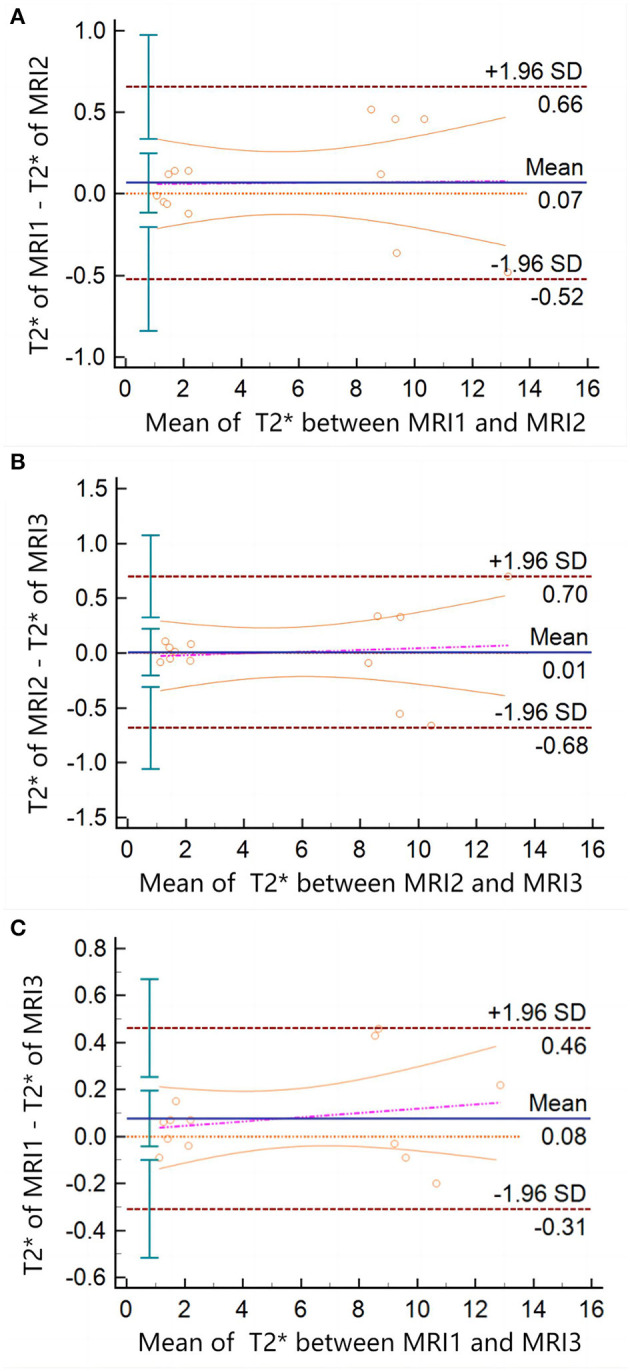
Bland–Altman plots of T_2_^*^ measured between different MRI scanners. There is no data point exceeding 95%CI in **(A, B)**, or panel **(C)**. The arithmetic mean between the T_2_^*^ values measured by the different MRI scanners was 0.06769 (*P* = 0.4323; 95%CI = −0.1138 to 0.2492), 0.009231 (*P* = 0.9264; 95%CI = −0.2040 to 0.2225), and 0.07692 (*P* = 0.1832; 95%CI = −0.04173 to 0.1956), respectively.

The intra-observer ICC calculated based on the two measurements of observer A was 0.996 (95% confidence interval (CI) = 0.992–0.998) and *P* < 0.001. The inter-observer ICC between observers A and B was 0.978 (95%CI = 0.940–0.990), and *P* < 0.001. The ICC between different MRI scanners was 0.999 (95%CI = 0.998–1) and *P* < 0.001. The results showed that the T_2_^*^ values obtained by different MRI scanners were highly consistent with intra-observer and inter-observer. The Bland–Altman plots also showed significant consistency in the T_2_^*^ values measured by different MRI scanners (Figure 3).

### 3.3. Formula construction

**Figure 4:**
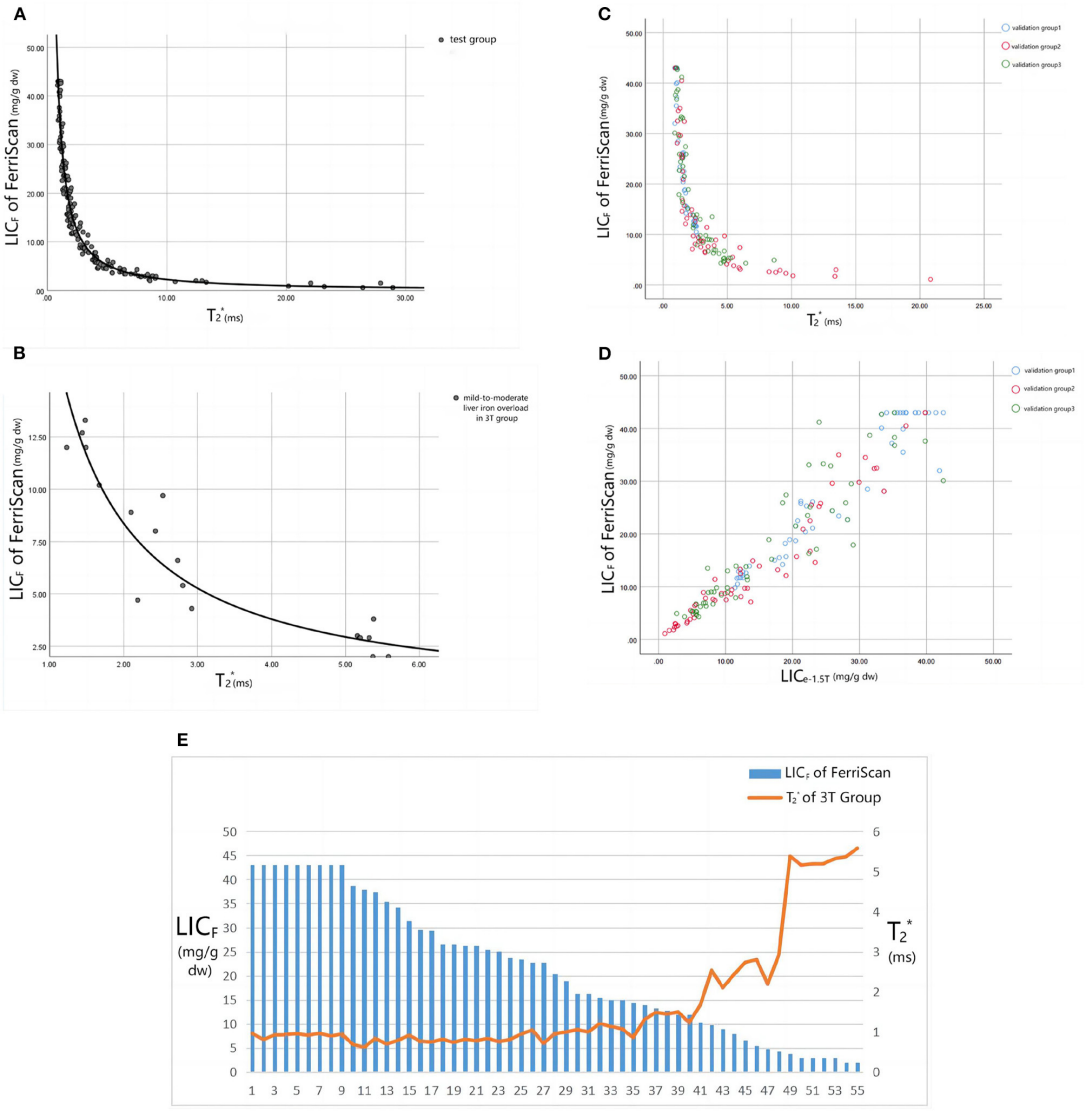
**(A)** Shows the fitting curve of LIC_F_ and 1.5T T_2_^*^ value in the test group, and the fitting equation is LIC_F_ = 37.393${\text{T}}_{2}{}^{*\wedge }$(−1.22) (R^2^ = 0.971, *P* < 0.001). **(B)** Shows the fitting curve of LIC_F_ and 3T T_2_^*^ value for mild-to-moderate iron overload in the 3T group, and the fitting equation is LIC_F_ = 18.463T_2_* ∧ (−1.142) (R^2^ = 0.889, *P* < 0.001). **(C, D)** Show the trend scatter plots of the relationship between LIC_F_ and 1.5T T_2_^*^ and LIC_F_ and LIC_e − 1.5T_ values in each validation group. **(E)** Shows the trend plot of the relationship between LIC_F_ and 3T T_2_^*^ values in the 3T group: when LIC_F_ > 14 mg/g dw, the T_2_^*^ value remained approximately 0.9 ms (the scanner could not accurately quantify the liver iron load in the range of LIC_F_ > 14 mg/g dw). Only when LIC_F_ < 14 mg/g dw did T_2_^*^ values displayed a negative relationship with LIC_F_.

The trends of the relationships between LIC_F_ and T_2_^*^ and LIC_F_ and LIC_e_ in each group are shown in Figure 4. According to the T_2_^*^ value of the test group and LIC_F_, the curve equation constructed was LIC_F_ = 37.393${\text{T}}_{2}{}^{*\wedge }$(−1.22) (R^2^ = 0.971, *P* < 0.001) (Figure 4A). According to the T_2_^*^ value of the 3T group and LIC_F_ (< 14 mg/g dw) of mild-to-moderate iron overload, the curve equation was LIC_F_ = 18.463${\text{T}}_{2}{}^{*\wedge }$(−1.142) (R^2^ = 0.889, *P* < 0.001) (Figure 4B). The liver T_2_^*^, LIC_F_, LIC_e − 1.5T_, and LIC_e − 3T_ values, and statistical description indicators of thalassemia patients in each group are shown in Table 1.

**Table 1:** Statistical descriptive indicators of liver T_2_^*^, LIC_F_, LIC_e − 1.5T_, and LIC_e − 3T_ values of thalassemia patients in each group.

**Group**	**Variable name**	**Number (N)**	**Min. ~maximum value**	**Quartile (P_25%_)**	**Quartile (P_75%_)**	**Median (M)**
Test group	T_2_^*^ (ms)	191	0.86–28.92	1.19	4.25	2
	LIC_F_ (mg/g dw)	191	0.60–43.00	5.7	29.5	14.9
Validation group1	T_2_^*^ (ms)	82	0.90–25.44	1.0875	4.4375	1.85
	LIC_F_ (mg/g dw)	82	0.90–43.00	6.025	35.925	14.6
	LIC_e − 1.5T_ (mg/g dw)	82	0.72–42.52	6.08	33.755	17.6
Validation group2	T_2_^*^ (ms)	54	0.95–20.83	1.5075	5.155	2.625
	LIC_F_ (mg/g dw)	54	1.10–43.00	5.325	23.175	9.55
	LIC_e − 1.5T_ (mg/g dw)	54	0.92–39.81	5.2751	22.9435	12.1968
Validation group3	T_2_^*^ (ms)	64	0.90–28.92	1.4725	4.76	2.885
	LIC_F_ (mg/g dw)	64	0.60–43.00	5.225	24.925	9.8
	LIC_e − 1.5T_ (mg/gdw)	64	0.62–42.52	5.2751	23.3285	10.2663
3T group (mild-to-moderate range)	T_2_^*^ (ms)	19	1.23–5.58	1.67	5.2	2.73
	LIC_F_ (mg/g dw)	19	2.00–13.30	2.9	10.2	5.4
	LIC_e − 3T_ (mg/g dw)	19	2.59–14.58	2.81	10.28	5.86
3T group (moderate-to-severe range)	T_2_^*^ (ms)	46	0.61–2.80	0.8	1.2075	0.94
	LIC_F_ (mg/g dw)	46	5.40–43.00	14.375	37.55	24.4
	LIC_e − 3T_ (mg/g dw)	46	5.70–32.47	14.8875	23.82	19.81

### 3.4. Formula verification

**Figure 5:**
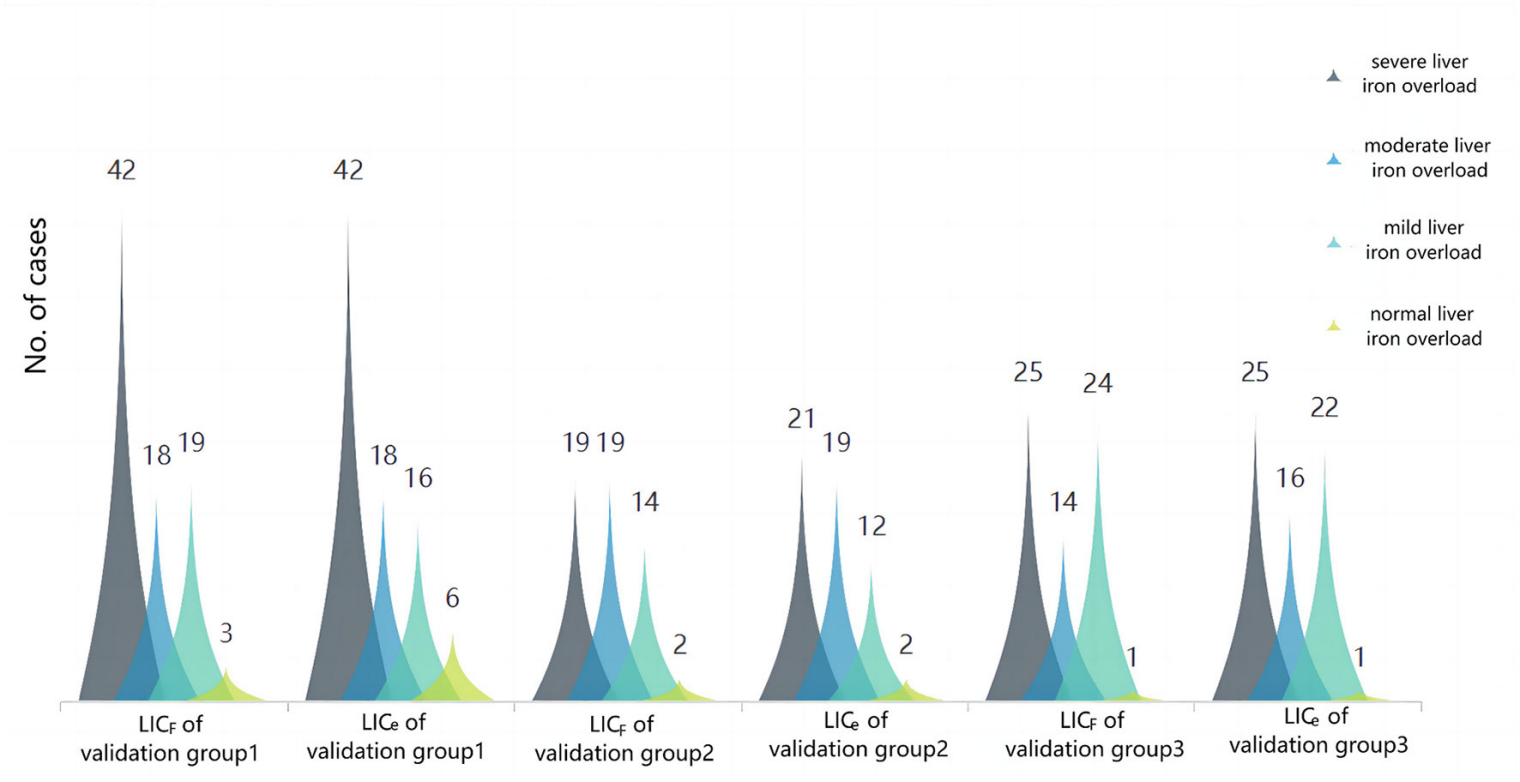
Cluster plot of the constituent ratio of clinical grades of liver iron overload in LIC_F_ and LIC_e − 1.5T_ for each validation group. The results showed that the overall distribution of the two methods was almost the same, and there were slight differences in some distributions: in validation group 1, three patients (3.66%) with liver iron overload were classified as mild according to LIC_F_ classification, and as normal according to LIC_e_ classification. In validation group 2, two patients (3.70%) had liver iron overload classified as mild according to LIC_F_ and moderate according to LIC_e_. In total, two patients (3.70%) had moderate liver iron overload according to LIC_F_ and severe liver iron overload according to LIC_e_. In validation group 3, two patients (3.13%) had mild liver iron overload according to LIC_F_ classification and moderate liver iron overload according to LIC_e_ classification.

There was no significant difference between LIC_e − 1.5T_ and LIC_F_ in verification groups 1, 2, and 3 (Z = −1.269, −0.977, −1.197; *P* = 0.204, 0.328, 0.231). There was significant consistency between them (Kendall's *W* = 0.991, 0.985, 0.980; *P* < 0.001) and high correlation (r_s_ = 0.983, 0.971, 0.960; *P* < 0.001). There was no statistical difference between the clinical grading results of LIC_e − 1.5T_ and LIC_F_ in verification groups 1, 2, and 3 (χ^2^ = 3.0, 4.0, 2.0; *P* = 0.083, 0.135, 0.157), and the grading results are shown in Figure 5. There was significant consistency among the clinical grading results (Kappa's K = 0.943, 0.891, 0.953; *P* < 0.001). The accuracy indexes and corresponding cutoff values of the clinical classification results of LIC_e − 1.5T_ for the test group and each validation group are shown in Table 2.

**Table 2:** Accuracy indicators and clinical classification cutoff values of LIC_e − 1.5T_ clinical classification results in the validation group.

**Group**	**Liver iron overload grading**	**AUC**	**95%CI**	***P***	**Specificity**	**Sensitivity**	**Youden index**	**Cut-off values (mg/g dw)**
Test group-LIC_1.5T_	Severe vs. moderate groups	0.991	0.983–0.999	< 0.0001	0.967	0.94	0.907	16.005
	Moderate vs. mild groups	0.942	0.904–0.980	< 0.0001	0.929	1	0.929	7.49
	Mild vs. normal group	0.999	0.995–1.000	< 0.0001	0.989	1	0.989	2.3
Validation group 1-LIC_1.5T_	Severe vs. moderate groups	1	1.000–1.000	< 0.0001	1	1	1	15.425
	Moderate vs. mild groups	0.969	0.927–1.000	< 0.0001	0.937	1	0.937	7.485
	Mild vs. normal group	0.988	0.963–1.000	0.019	0.987	1	0.987	1.225
Validation group 2-LIC_1.5T_	Severe vs. moderate groups	0.995	0.985–1.000	< 0.0001	1	0.947	0.947	19.814
	Moderate vs. mild groups	0.95	0.985–1.000	< 0.0001	0.95	1	0.95	6.123
	Mild vs. normal group	1	0.963–1.000	0.019	0.981	1	0.981	2.302
Validation group 3-LIC_1.5T_	Severe vs. moderate groups	1	1.000–1.000	< 0.001	0.974	1	0.974	13.22
	Moderate vs. mild groups	0.972	0.923–1.000	< 0.001	0.975	0.958	0.933	7.199
	Mild vs. normal group	1	1.000–1.000	0.008	0.984	1	0.984	2.182
3T group-LIC_3T_	Severe vs. moderate groups	0.997	0.988–1.000	< 0.001	1	0.972	0.972	14.785
	Moderate vs. mild groups	0.977	0.920–1.000	< 0.001	0.909	1	0.909	6.13

**Figure 6:**
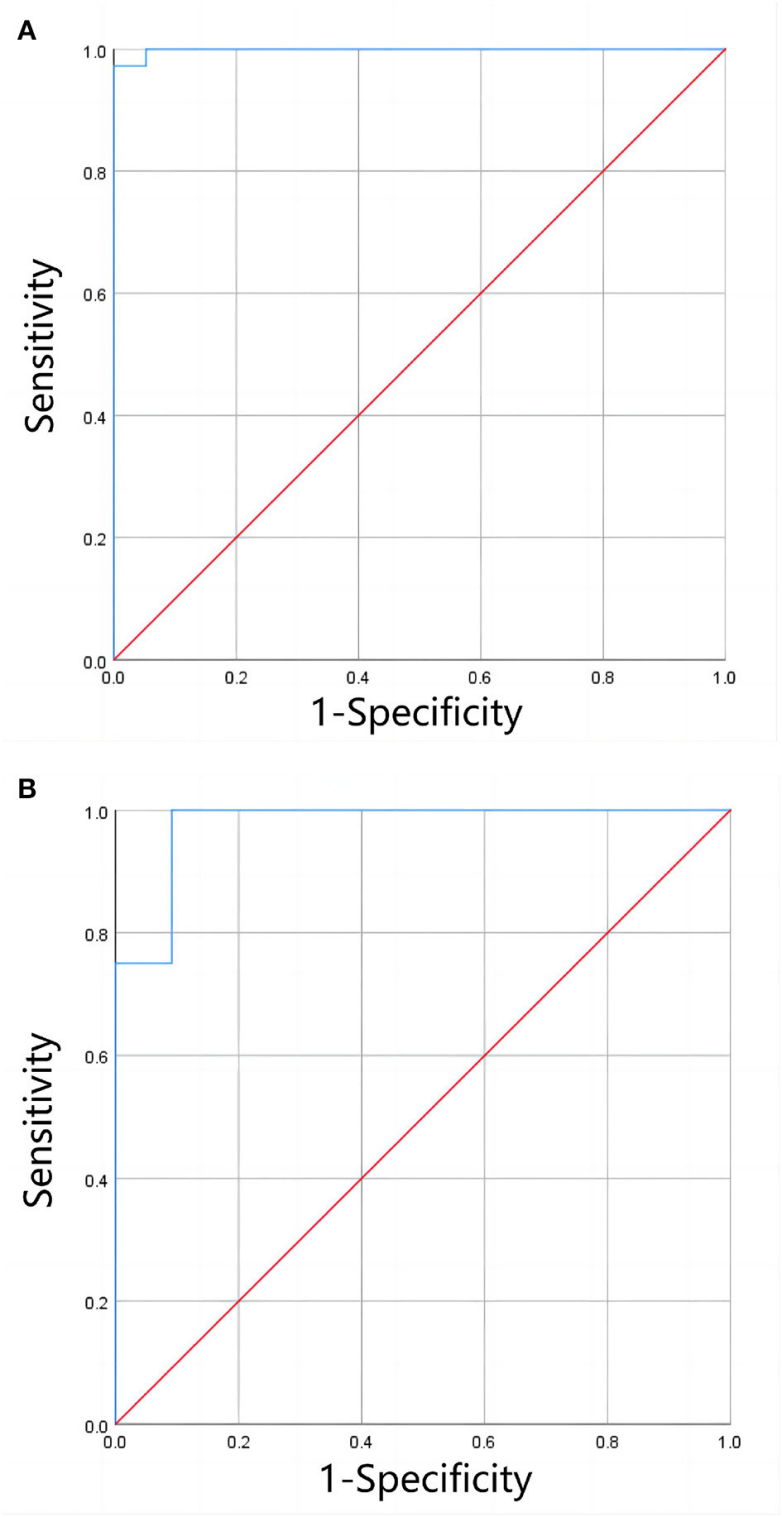
**(A, B)** Shows the ROC curves for clinical grading of moderate and severe liver iron overload, and of mild and moderate liver iron overload by LIC_e − 3T_, respectively. The results showed that LIC_e − 3T_ had a high diagnostic efficiency for clinical grading of liver iron overload when only clinical grading diagnosis was performed (without precise quantification of LIC).

In the 3T group, LIC_F_ in the mild-to-moderate range was significantly correlated with the corresponding T_2_^*^ value (r_s_ = −0.940, *P* < 0.001). There was no statistically significant correlation between LIC_F_ and T_2_^*^ values in the severe range (*P* = 0.085). Of the 36 patients with severe iron overload diagnosed by LIC_F_, 1 (2.78%) was diagnosed with moderate iron overload by LIC_e − 3T_. Of the eight patients with moderate iron overload diagnosed by LIC_F_, two (25%) were diagnosed with mild iron overload by LIC_e − 3T_. Of the 11 patients with mild iron overload diagnosed by LIC_F_, one (9.10%) was diagnosed with moderate iron overload by LIC_e − 3T_. There was no significant difference between LIC_F_ and LIC_e − 3T_ in the mild to moderate range (Z = −0.523, *P* = 0.601). There was a significant correlation between them (r_s_ = 0.940, *P* < 0.001) and significant consistency (Kendall's *W* = 0.970, *P* = 0.008). There was no significant difference between the clinical grading results of full range LIC_F_ (χ^2^ = 1.333, *P* = 0.513) and LIC_e − 3T_ (χ^2^ = 1.333, *P* = 0.513). There was significant consistency among the clinical grading results (Kappa's K = 0.860, *P* < 0.001). The ROC curve of LIC_e − 3T_ clinical grading results is shown in Figure 6, and the evaluation indices of diagnostic accuracy are shown in Table 2.

## 4. Discussion

As mentioned, magnetic resonance imaging has been widely considered the primary method for the non-invasive determination of liver iron concentration (6). FerriScan based on R_2_ technology can generate reports including liver iron concentration, but it cannot be widely applied due to a variety of limiting factors, especially for long-term and timely quantitative monitoring of LIC in patients (16). T_2_^*^/R_2_^*^ image-based relaxometry and related measurement techniques have been developed in many centers. After years of research, many scholars have verified that the T_2_^*^/R_2_^*^ value has an obvious linear relationship with LIC and partially constructed the curve equation of the relationship between them (8–13).

In the first quantitative study of liver iron overload using R_2_^*^ relaxation measurement by Henninger et al. (8), the relevant parameters set were repetition time (TR) = 200 ms and initial echo time (TE) = 0.99 ms. Liver biopsy and MRI were performed on 17 patients with clinical suspicion of liver iron overload. The final regression model between R_2_^*^ and LIC was constructed as follows: LIC = 0.024R_2_^*^ + 0.277, correlation coefficient = 0.926, slope = 0.024 (mg/g) [95% CI = 0.013–0.024], intercept = 0.277 (mg/g) [95% CI = 0.328–2.49]. In an early study by Wood et al. (9), the set TE was increased from the initial 0.8 ms to 4.8 ms at 0.25 ms intervals in a breath hold, and TR = 25 ms. After MRI evaluation of 102 patients with liver iron overload (the biopsy-measured LIC was evenly distributed between 1.3 mg/gdw and 32.9 mg/gdw, and one patient had a HIC of 57.8 mg/gdw), the final LIC-R_2_^*^ regression equation was constructed as follows: the correlation coefficient was 0.97, the slope was 37.4 Hz/mg/gdw, and the y-intercept was 23.7 Hz. In an early study by Hankins et al. (10), TE = 1.1–17.3 ms (20 echoes) was set, and 43 patients (32 with sickle cell anemia, six with major β-thalassemia, five patients with bone marrow failure) underwent MRI examination and liver biopsy (LIC range = 0.6 mg Fe/g to 27.6 mg Fe/g). The final LIC-R_2_^*^ regression model was constructed as follows: the intercept was −454.85, the slope was 28.02 (*P* < 0.001), the R^2^ was 0.72, and the correlation coefficient was 0.98. In an early study by Christoforidis et al. (11), MRI was performed on 94 patients with β-thalassemia major with TE = 2.24–20.13 ms and TR = 200 ms. The relationship between liver–muscle ratio (MRI-LIC = 5–350 μmol/g) and R_2_^*^ (27.03–1,298.70 s^−1^) was compared. The final LIC-R_2_^*^ regression model was R_2_^*^ = 0.851(MR-LIC) – 2.137 (correlation coefficient = 0.851). In the study by Garbowski et al. (12), TE was set as 0.93–16.0 ms. A total of 54 patients (36 cases of thalassemia major, seven cases of sickle cell anemia, four cases of myelodysplastic syndrome, three cases of Diamond-Blackfan anemia, two cases of red cell aplasia, two cases of pyruvate kinase deficiency anemia), and 31 healthy volunteers underwent liver biopsy (LIC = 1.7–42.3 mg/g dw) and MRI examination (R_2_^*^ range = 28.7–54.4 s^−1^). The final regression models of LIC (biopsy) -T_2_^*^ and LIC (biopsy)-R_2_^*^ were constructed: (1) LIC = 31.94(T_2_^*^)^−1.014^, 95%CI of coefficient = 27.8–36.7 (87–115%), 95%CI of index = −1.118–0.91(110–90%). (2) LIC = 0.029(R_2_^*^)^1.014^, 95%CI of coefficient = 0.016–0.054 (55–186%), 95%CI of index = 0.910–1.118 (90–110%). Garbowski et al. (12) also constructed the correction relationship between LIC (Ferriscan)-R_2_^*^ and LIC-T_2_^*^: (1) R_2_-LIC = 0.83T_2_^*^ – LIC^1.04^, 95%CI of coefficient = 0.96 ~ 1.11, 95%CI of index = 0.55 ~ 1.29. (2) R_2_-LIC = 0.87R_2_^*^ – LIC – 0.55, 95%CI of slope = 0.74–0.99, 95%CI of intercept = −0.01–1.19.

In this study, based on large sample size, multicenter validation, and complete statistical analysis, the equation LIC_F_ = 37.393${\text{T}}_{2}{}^{*\wedge }$ (−1.22) was proposed to quantify LIC from 1.5T MRI T_2_^*^. For the 3T MRI quantification of liver iron overload, the relationship LIC_F_ = 18.463${\text{T}}_{2}{}^{*\wedge }$(−1.142) was proposed to quantify LIC in patients with mild-to-moderate iron overload. However, it is still not possible to accurately quantify LIC in patients with severe liver iron overload at 3T field strength. This also suggests that the 3T T_2_^*^ technique should be avoided for the quantification of LIC in patients with severe iron overload, and 1.5T or other methods should be used instead. This conclusion is similar to that of d'Assignies (15). It is worth noting that this study found that when 3T T_2_^*^ technology was used to quantify liver iron overload, although the T_2_^*^ value of patients with severe liver iron overload was almost maintained at 0.9 ms, it was impossible to further accurately quantify the LIC value; however, if only the clinical classification of liver iron overload was performed, that is, only the classification of mild-to-moderate and severe iron overload was performed, the classification of liver iron overload would have high diagnostic efficacy.

The slope of the calibration curve proposed by different studies is different, and for the LIC calculated by the earlier calibration curve, Garbowski et al. (12) also proposed further calibration coefficients to calibrate the final LIC. The specific reasons for the differences are analyzed as follows: (1) the previous T_2_^*^/R_2_^*^-LIC calibration curve equation was based on liver biopsies, such as the study by Henninger et al. (8), Wood et al. (9), Hankins et al. (10), Christoforidis et al. (11), and Garbowski et al. (12). Although LIC provided by liver biopsy has been used as the “gold standard” for a long time, the materials and methods used in the process of liver biopsy, and the heterogeneity of iron in the liver will lead to differences between different studies; (2) The sample size used in some studies is small. A small sample size will not only increase the sampling error, but also limit the range of LIC used, and the final fitted calibration curve equation cannot be extended to quantify a wider range of LIC. For example, the LIC of 17 patients collected by Henninger et al. (8) through liver biopsy ranged from 0.917 mg/g to 11.646 mg/g. The authors believe that because the range of LIC studied is small, it is not appropriate to use the corresponding calibration curve to quantify a wider range of LIC; (3) Different models used to measure T_2_^*^/R_2_^*^ will directly lead to differences in the final LIC. For example, Wood et al. (9) used an offset model, while Garbowski et al. (12) used a truncated model to measure R_2_^*^. Garbowski et al. (12) proposed that the R_2_^*^ value measured by the offset model was high, while the R_2_^*^ value measured by the truncated model was low, and emphasized the importance of using the appropriate measurement model to quantify T_2_^*^/R_2_^*^ and the appropriate analysis techniques to construct the curve equation in clinical practice; (4) The high iron concentration corresponds to a very low T_2_^*^ value, thus it is necessary to set a very short minimum echo time for more accurate measurement. However, due to the differences in the technique and scanning sequence used by different research centers, the different minimum echo time set obviously limits the lowest T_2_^*^ value, which is the maximum value of LIC measured by the center. Some studies have shown that LIC with severe iron overload in the liver should be measured in combination with the signal intensity ratio between the liver and the paravertebral muscles (SIR) (17).

The shortcomings of this experiment are as follows: (1) the definition of the ROI. In this study, the delineated T_2_^*^ image ROI was as close as possible to the T_2_ image ROI delineated by FerriScan, but the artificial delineation of ROI was susceptible to various subjective and objective factors, and measurement error was inevitable; (2) In this study, the proportion of patients with moderate or severe liver iron overload was relatively large, and the proportion of patients with mild iron concentration was relatively small, which had a certain bias. However, in general, this study was analyzed with large sample size and was validated in multiple centers, which makes the results reliable; (3) In this retrospective study, LIC_F_ was used as the reference standard, in other words, the T_2_^*^-LIC calibration equation was constructed under the assumption that FerriScan based on T_2_/R_2_ technique was very reliable. Therefore, the equations obtained should not be extended to other techniques or organs for calculating iron concentration; (4) The sample size of 3T data is small, and it is difficult to perform grouping verification; (5) Accurate quantification of severe liver iron load by 3T T_2_^*^ technique still cannot be achieved due to the technical limitation of the 3T scanning sequence in this study.

## 5. Conclusion

This study explored the relationship between liver T_2_^*^ value and LIC_F_ provided by FerriScan in patients with thalassemia, and the related curve equation was constructed. After measuring the liver 1.5T T_2_^*^ value, the liver iron concentration could be accurately quantified, and liver iron concentration in patients with iron overload could be better monitored. An important reference for timely and better formulation of appropriate diagnosis and treatment plans can be made. 3T T_2_^*^ can be used to quantify liver iron concentration in patients with mild-to-moderate liver iron overload, while 1.5T T_2_^*^ or other methods are recommended for patients with severe liver iron overload.

## Data availability statement

The original contributions presented in the study are included in the article/supplementary material, further inquiries can be directed to the corresponding author.

## Ethics statement

The studies involving human participants were reviewed and approved by the Ethics Committee of the First Affiliated Hospital of Guangxi Medical University (2022-E457-01). Written informed consent from the participants' legal guardian/next of kin was not required to participate in this study in accordance with the national legislation and the institutional requirements.

## Author contributions

PP contributed to the study's conception and design. Materials preparation and data collection were performed by FX, YP, HX, GY, FZ, and YL. Data analysis was performed by FX, HX, and YP. The first draft of the manuscript was written by FX and BL and all authors commented on earlier drafts of the manuscript. All authors have read and approved its final version.
